# Association of *Trichomonas vaginalis* and Cytological Abnormalities of the Cervix in Low Risk Women

**DOI:** 10.1371/journal.pone.0086266

**Published:** 2013-12-30

**Authors:** Gilbert G. G. Donders, Christophe E. Depuydt, John-Paul Bogers, Annie J. Vereecken

**Affiliations:** 1 Departments of Obstetrics and Gynecology, University Hospital Antwerpen, Antwerpen, Belgium; 2 Department of Obstetrics and Gynecology, Heilig Hart Regional Hospital, Tienen, Belgium; 3 Department Clinical Research for Women, Tienen, Belgium; 4 Laboratory for Molecular and Clinical Pathology (RIATOL), AML Laboratory Sonic Healthcare Benelux, Antwerp, Belgium; University of Toronto, Canada

## Abstract

**Objective:**

Is *Trichomonas vaginalis* (TV) an inducing factor for the development of (pre-)cancerous lesions of the cervix?

**Design:**

Cross sectional study.

**Setting:**

Screening healthy Belgian women with low infection risk.

**Sample:**

63,251 consecutive liquid based cervical samples.

**Methods:**

Real time quantitative PCR for presence of TV, 18 HPV types and Pap smear analysis of cytologic abnormalities.

**Main Outcome Measures:**

Association of TV and HPV with cervix dysplasia

**Results:**

The overall prevalence of TV DNA was 0.37%, of low risk HPV 2%, of high risk HPV 13.2%, and 8.8 % had cytological abnormalities. Both LR-HPV and HR-HPV were significantly associated with all cytological abnormalities. Presence of TV was associated with LR- and HR-HPV, ASC-US and HSIL, but not with other abnormalities. All women with TV and HSIL also had HR-HPV, while the latter was present in only 59% of women with TV and ASC-US. Amongst HPV negative women, TV was found in 1.3% of women with ASC-US, but only in 0.03% of women with normal cytology (OR 4.2, CL95% 2.1-8.6). In HR-HPV positive women, presence of TV increased the likelihood of cytological abnormalities somewhat (P=0.05), mainly due to an increase in ASC-US and LSIL, but not HSIL.

**Conclusions:**

We conclude that TV infection is associated with both LR and HR-HPV infection of the cervix, as well as with ASC-US and HSIL. TV is a concomitant STI, but is not thought to be a co-factor in the causation of HSIL and cervical cancer. However, TV may cause false positive diagnoses of ASC-US.

## Introduction

Both human papilloma virus (HPV) and *Trichomonas vaginalis* (TV) infection are common sexually transmitted infections (STIs) worldwide [1]. The increased risk of coexistent infections once a first STI is diagnosed is well known and leads to the recommendation to test for other genital infections simultaneously [2]. Despite this, it is not uncommon for women with HPV infections, nowadays readily detected by extended testing for cervical dysplasia and cancer, not to be tested for other STIs. TV is an under-recognized condition despite its clinical importance [3-5], probably due to its perceived rarity, the additional diagnostic effort requiring a fresh specimen for microcopy and/or culture, and its largely asymptomatic appearance in men and women [6,7]. Introduction of polymerase chain reaction (PCR)-based detection techniques for TV, 5 low-risk (LR) and 13 high-risk (HR) HPV types allowed us to determine the prevalence of these infections in our population and its relationship with HPV infection [8]. In some studies, TV is depicted as a risk factor for cervical dysplasia and cancer [9]. We assessed the possible role of TV in the pathogenesis of such precancerous lesions of the cervix. 

## Methods

### Study population and used tests

All clinical cervico-vaginal samples, received between 15^th^ April and 1^st^ November 2008 by the Laboratory for Clinical Pathology (labo RIATOL), Antwerp, Belgium, for cytologic examination and TV testing, were tested for presence of *Trichomonas vaginalis* and HPV DNA. The medical ethics committee of the University of Antwerp confirmed that ethical oversight is unnecessary for retrospective case studies such as this.  All data were anonymized, no specific tests were performed outside routine practice, and there was no cost or additional risk to the patient.

Cervico-vaginal cells were collected with the Cervex-Brush Combi (Rovers Medical Devices B.V., Oss, The Netherlands). After collection, brush heads were transferred directly into a vial with BD-SurePath preservative fluid [10]. From the fluid containing the cellular material, a liquid based cytology sample was prepared with the robotic BD PrepStain Slide Processor, previously AutoCyte PREP System (BD Diagnostics - TriPath, Burlington, NC, USA) [11,12]. All slides are prescreened using BD-FocalPoint, a computerized scanning system for the primary screening of cervical smears (BD Diagnostics), followed by targeted microscopic interpretation of selected suspicious fields using BD-FocalPoint guided screening review stations. Cytology results were classified according to the Bethesda system 2001 [13,14] thus: negative for intraepithelial lesions and malignancies (NILM); atypical squamous cells of undetermined significance (ASC-US); atypical glandular cells (AGC); low-grade squamous intraepithelial lesions (LSIL); atypical squamous cells of undetermined significance, cannot exclude high-grade squamous intraepithelial lesions (ASC-H); and high-grade squamous intraepithelial lesions (HSIL). DNA isolation from the cellular pellet remaining after processing of the BD SurePath™ cytology specimen was performed as previously described [14,15]. The presence of HPV genotypes and *Trichomonas vaginalis* was determined using a multiplex TaqMan-based real-time quantitative PCR. The sequences for the primers and probes used for different type specific HPV qPCRs can be found in the paper by Micalessi and coworkers [16]. For detection of HPV genotypes type-specific sequences of viral E6 or E7 genes: 6 E6, 11 E6, 16 E7, 18 E7, 31 E6, 33 E6, 35 E6, 39 E7, 45 E7, 51 E6, 52 E7, 53 E6, 56 E7, 58 E6, 59 E7, 66 E6, and 68 E7 were targeted [14,15]. HPV types 6, 11, 53, 66 and 67 were also detected but were not considered as high-risk (LRHPV). The target for detection of TV was a 67-base pair region of a repeated sequence of the TV genome (Gene Bank Accession Number L23861) [8,18,19]. Multiple validation tests under different conditions were done with 15 clinical samples with proven TV, and 150 proven negative samples before commencing large scale testing. 

The analytic sensitivity of the different PCR assays ranged from 1 to 100 copies and was calculated using standard curves for 16 type-specific PCRs constructed with plasmids containing the entire genome of the different HPV types [15]. Real-time quantitative PCR for ß-globin was used to verify the quality of DNA in the sample and to measure the amount of input DNA [15,17].

### Statistical analysis

Statistical analysis was performed with Chi-squared or Fisher exact test when appropriate for absolute numbers, and Student t test for normal distributed continuous variables.

## Results

From 15th April to 1st November 2008, 64241 consecutive cervical cytology samples were processed in the laboratory. We excluded 966 (1.50%) follow up samples and 24 (0.04%) samples in which no human ß-globin DNA could be amplified.

For 63,251 (98.5%) of the remaining samples cytology, HPV and TV results were available (Table **1**). The frequency of LRHPV was 2.0% (1234/63251) and of HRHPV infection 13.5% 8346/63251). TV was detected in 236 (0.37%). 2243 (3.5%) had atypical squamous cytologic abnormalities of unknown significance (ASC-US), 111 (0.2%) atypical glandular cytologic abnormalities (AGC), 2310 ( 3.6%) low grade squamous intraepithelial lesion (LSIL), 243 (0.4%) atypical squamous cytologic abnormalities of high grade potential (ASC-H) and 660 (1.0%) had a high grade squamous intraepithelial lesion (HSIL). 42 of the 236 TV positive samples had a abnormal cytology (ASC-US+, 17.8%), compared to 5525 of the 63251 TV negative samples (8.7%) (OR 2.3, CI 95% 1.6-3.2, p<0.0001). Significantly more women with ASC-US had TV (1%) than women with normal cytology (0.34%) (OR 2.9, CI95% 1.9-4.6, p<0.0001). Similar figures where found for HSIL, where TV was also found significantly more frequently (1% vs 0.34%, OR 3.2, CL95%1.5-6.8, p=0.0047). In both AGC and ASC-H groups no TV positive samples were detected (Table **1**). 

**Table 1:** Prevalence of *Trichomonas vaginalis* according to human papilloma virus (HPV) status and cervix cytology group.

		NILM	ASCUS	AGC	LSIL	ASCH	HSIL	Total
HPV negative	N	52714	620	64	228	18	27	53671
	TV+	168	8^11^	0	2	0	0	178
	(%)	(0,32)	(1,29)	(0)	(0,88)	(0)	(0)	(0.33)
LR-HPV positive	N	842	160^1^	5^2^	197^3^	8^4^	22^5^	1234
	TV+	10^12^	1	0	0	0	0	11
	(%)	(1,19)	(0,63)	(0)	(0)	(0)	(0)	(0.88)
								OR 2.7 (1.5-5.0) P=0.0023
HR-HPV positive	N	4128	1463^6^	42^7^	1885^8^	217^9^	611^10^	8346
	TV+	16	13	0	11	0	7	47
	(%)	(0,39)	(0,89)	(0)	(0,58)	(0)	(1,15)	(0.55)
								OR 1.7 (1.2-2.3) P=0.0015
Total in each group	N	57684	2243	111	2310	243	660	63251
Total TV+	N	194	22	0	13	0	7	236
	(%)	(0,34)	(0,98)	(0)	(0,56)	(0)	(1,06)	(100)
		Ref	OR 2.9 (1.9-4.6) P<0.0001	Ns	Ns	Ns	OR 3.2 (1.5-6.8) P=0.0047	

Data are given as absolute numbers and percentages (in italics). HR-HPV (high risk human papilloma virus), positive for one of the following types: 16, 18, 31, 33, 35, 39, 45, 51, 52, 56, 58, 59 and 68; LR-HPV (low risk human papilloma virus), positive for one of the following types: 6, 11, 53, 66 and 67; TV: *Trichomonas vaginalis*. N: total number. NILM: negative for intraepithelial lesions and malignancies, ASCUS: atypical squamous cells of undetermined significance, AGC: atypical glandular cells, LSIL: low-grade squamous intraepithelial lesions, ASC-H: atypical squamous cells of undetermined significance, but cannot exclude high-grade squamous intraepithelial lesions and HSIL: high-grade squamous intraepithelial lesions. Statistical significance in different cytology groups between women negative for HPV and positive for:

a) low risk HPV infection: (1) OR 19.7 (16.3-23.8) P<0.0001 ; (2) OR 4.9 (1.9-12.2) P<0.000; (3) OR 70.3 (57.2-86.4) P<0.0001; (4) OR 28.1 (12.2-64.8) P<0.0001; (5) OR 52.4 (29.7-92.3) P<0.0001

b) high risk HPV infection: (6) OR 46.1 (41.7-51.1) P<0.0001; (7) OR 8.5 (5.7-12.5) P<0.0001 ; (8) OR 193.5 (167.5-223.4) P<0.0001; (9) OR 162.4 (100.3-263.0) P<0.0001; (10) OR 339.0 (230.2-499.2) P<0.0001

*Trichomonas vaginalis* infection: (11) OR 4.2 (2.1-8.6) P<0.0001; (12) OR 3.8 (2.0-7.1) P<0.0001

When studying the correlation of LR and HR HPV and cytological abnormalities we found evidence of HPV infection in 7.8% in women without cytological abnormalities, and in 65.2%, 31.8%, 81.6%, 89.3% and 92.6% of women with ASC-US, AGC, LSIL, ASC-H and HSIL respectively. Both LR HPV and HR HPV were significantly associated with all cytologic abnormalities (ASCUS, ASC-H, AGC, LSIL and HSIL, p<0.0001, Table **1**). TV infection was correlated with both LR HPV (OR 2.7, CL95% 1.5-5.0) and with HR HPV (OR1.7, CL95% 1.2-2.3). All women with TV and HSIL had HR HPV, while HR HPV was only present in 59% of women with TV and ASC-US. 

In the HPV negative ASC-US group TV was detected in 1.3% (8/620), compared to 0.86% (14/1623) in the HPV positive (LR+HR) ASC-US group (OR 1.5, CL95% 0,63 - 3,6, p=0.36).

Amongst all HPV negative women, TV was found in 1.3% with ASCUS, and 0.03% with normal cytology (OR 4.2, CL95% 2.1-8.6, p<0.0001). 

In HR HPV positive patients, the likelihood of having ASC-US+ was slightly higher when TV was present than when TV was absent (31/47 vs 4218/8346, OR1.98 CL95% 1.04-3.5, P=0.049). However, the majority of lesions in the group with combined HR HPV/TV infections were ASC-US (13/31) or LSIL (11/31) and not HSIL (7/31). 

## Discussion

In this study, we confirm a correlation of TV with HPV and with cytological abnormalities of the cervix, indicating that STIs are often coexist, even in a setting where the prevalence of TV is very low. This low prevalence makes the interpretation of the role of TV in the pathogenesis of cytologic abnormalities less evident in certain subgroups, despite high numbers of samples examined, a reliable diagnostic technique used (Real time PCR) and the completeness of the data obtained, which all add to the strength of the study. 

The correlation of LR HPV and HR HPV with different types of cytological abnormalities confirms expected HPV detection rates in cervical dysplastic lesions, as discussed in other studies. The prevalence of *T. vaginalis* is low in Flanders at 3.7 per 1000. Furthermore, we found that the age-dependent prevalence and epidemiology for HPV and TV were very different in this population, with a peak incidence of HPV infection at the age of 20-25, and of TV infection at 45-55 years [8], a finding repeated in several US centers [20,21]. Despite these differences, TV is clearly to be seen as an STI, as it is both correlated with HPV infection (both low and high risk) and with abnormal cytology of the cervix, a finding confirming evidence from other series [5,22,23]. As a consequence, some have speculated on the possible permissive role of TV in the pathogenesis of cervical intraepithelial lesions due to HPV [5,9]. 

With the present data, in a low prevalence setting, we could demonstrate that in all cases of HSIL where TV was discovered, HR HPV was also present as a causative agent. On the other hand, 36% (8/22) of the women with a combination of TV infection and ASC-US had no HPV. Although the prevalence of TV was higher in the HPV negative ASC-US group (1.3%) compared to the HPV positive (LR+ and HR+) ASC-US group (0.86%), this was not statistically significant. However since cytology reading was performed with knowledge of HPV status, presence of TV could still explain detection of atypical cells in the HPV negative ASC-US group. Also, in the group of HPV negative women, the infection rate of TV was 4 times higher in women with ASC-US than in cytological normal women. Furthermore, in HR HPV negative women, TV was never associated with HSIL or ASC-H, but was present in almost 1% of ASC-US and LSIL cases. These findings strongly suggest that TV can cause ASC-US like findings on cytology, without HR HPV or even any HPV present, and hence could lead to false positive diagnosis of ASC-US, enhancing the likelihood of overtreatment and unnecessary anxiety [24,25]. Other authors pointed out that TV can cause inadequate cytology readings, and pleaded for pretreatment of TV before performing cytology [26]. However, with the majority of TV infections being asymptomatic, and the accuracy of cytology in its detection being extremely poor and generally below 60% [27-29], the odds are high that presence of TV would continue to interfere with correct identification of low grade cellular atypia, especially ASC-US. Even taking into consideration an apparently better sensitivity for TV detection when liquid based media are used [30,31], it would be unlikely that cytology will enable TV detection in a sufficiently accurate way to prevent false positive readings, certainly not in high prevalence areas. Therefore we strongly suggest that real time PCR should be used to detect TV in the same sample used for cytology and HPV DNA detection.

Also, the apparent relation of TV with HSIL (OR 3.2) is almost certainly due to co- infection with HR HPV in all cases, instead of being causative or even a co-factor for the development of HSIL. As only 1% of all HSIL were harboring TV, it seems very unlikely that these sporadic TV would have a significant role in the pathogenesis of HSIL. Still, if present together with HR HPV infection, TV infection was associated with a slightly higher rate of ASC-US+ abnormalities than when only HR HPV were present. As 4 out of 5 of these ASC-US+ lesions were low grade lesions (ASC-US and LSIL), we suppose the small increase in HSIL in combined TV-HR HPV patients may also be due to non-specific cellular aberrations caused by TV, as we found in one third of ASC-US in TV infected women without HPV infection.

## Conclusion

We conclude that TV infection is associated with both low risk and high risk HPV infection of the cervix, as well as with the cervical cytological abnormalities ASC-US and HSIL. However, TV is unlikely to be involved in the causation or promotion of HSIL and cervical cancer. In women infected with TV, non-specific alterations of the epithelial cells may lead to a number of false positive diagnoses of ASC-US. In order to eradicate TV before making a final cytology diagnosis, we strongly advocate the use of real time PCR or systematic TV detection in liquid media before cytological examination. 
